# The poison oligonucleotide F10 is highly effective against acute lymphoblastic leukemia while sparing normal hematopoietic cells

**DOI:** 10.18632/oncotarget.1937

**Published:** 2014-05-01

**Authors:** Timothy S. Pardee, Kristin Stadelman, Jamie Jennings-Gee, David L. Caudell, William H. Gmeiner

**Affiliations:** ^1^ Department of Internal Medicine, Section on Hematology and Oncology, Wake Forest University School of Medicine, Winston-Salem, NC; ^2^ Comprehensive Cancer Center of Wake Forest University, Winston-Salem, NC; ^3^ Department of Cancer Biology, Comprehensive Cancer Center of Wake Forest University, Winston-Salem, NC; ^4^ Department of Pathology, Section of Comparative Medicine, Wake Forest University School of Medicine, Winston-Salem, NC

**Keywords:** Acute lymphoblastic leukemia, Fluoropyrimidine, novel therapy

## Abstract

F10 is an oligonucleotide based on the thymidylate synthase (TS) inhibitory 5-fluorouracil (5-FU) metabolite, 5-fluoro-2'-deoxyuridine-5'-O-monophosphate. We sought to determine the activity of F10 against preclinical models of acute lymphoblastic leukemia (ALL). F10 treatment resulted in robust induction of apoptosis that could not be equaled by 100 fold more 5-FU. F10 was more potent than Ara-C and doxorubicin against a panel of murine and human ALL cells with an average IC_50_ value of 1.48 nM (range 0.07 to 5.4 nM). F10 was more than 1000 times more potent than 5-FU. *In vivo,* F10 treatment significantly increased survival in 2 separate syngeneic ALL mouse models and 3 separate xenograft models. F10 also protected mice from leukemia-induced weight loss. In ALL cells made resistant to Ara-C, F10 remained highly active *in vitro* and *in vivo*. Using labeled F10, uptake by the ALL cell lines DG75 and SUP-B15 was rapid and profoundly temperature-dependent. Both cell lines demonstrated increased uptake compared to normal murine lineage- depleted marrow cells. Consistent with this decreased uptake, F10 treatment did not alter the ability of human hematopoietic stem cells to engraft in immunodeficient mice.

## INTRODUCTION

Acute lymphoblastic leukemia (ALL) is an aggressive hematologic malignancy wherein an abnormal proliferation of lymphoblasts suppresses normal hematopoiesis, resulting in progressive marrow failure and death [1]. There are approximately 6,000 cases per year diagnosed in the United States [1]. ALL has a bimodal age distribution with an initial peak in childhood and second that increases in older adults. While outcomes for ALL in children have improved (approximately 85-90% cure rate with modern chemotherapy regimens [2]), outcomes in adults are much worse (estimated 30-45% cure rate [3]). The cure rate is worse still in patients over the age of 60; the 5-year survival rate in these patients is estimated at ~10%, and has not significantly improved since the 1980's [3]. This is further complicated by the fact that the median age of adults at diagnosis is ≥60 [4].

This poor outcome is attributable to a combination of increased adverse tumor biology and decreased tolerance of therapy [5]. As a result, despite a high initial remission rate, most adults with ALL are destined to relapse, and once relapse occurs, outcomes are particularly dismal. In a large study of adult patients with ALL after their first relapse, the 5-year survival rate was 7%. The authors stated that most adults with recurrent ALL “cannot be rescued with current therapies” [6]. Virtually all active therapies are used during first-line treatment leaving nothing for relapsed disease. There is clearly a need for additional active therapies in ALL.

Many current drug development efforts are focused on the identification and targeting of specific deregulated oncogenic pathways. This is in part the result of the dramatic success of agents like imatinib that targets the BCR-ABL kinase. The use of imatinib in chronic-phase chronic myelogenous leukemia (CML) has changed the natural history of the disease [7]. Unfortunately, the durable responses seen in CML are not reproduced in BCR-ABL positive ALL [8]. Indeed, genetically homogenous cancers that are reliant on a single oncogenic pathway have proven to be the exception rather than the rule. Targeting of a single oncogenic pathway in genetically complex malignancies like ALL have resulted in only transient responses with frequent relapses.

An alternative approach is to use agents that target “final common pathways”, i.e. processes that must be accomplished to produce additional cancer cells regardless of driving mutations. In this paradigm, agents are not judged by differential expression of a target, but by the degree of differential uptake. One pathway to exploit is the known increased uptake of oligonucleotides by ALL cells [9]. F10 is a poison deoxy-oligonucleotide that is a 10mer of the TS inhibitory 5-FU metabolite, 5-fluoro-2'-deoxyuridine-5'-O-monophosphate. F10 has high activity against preclinical models of acute myeloid leukemia by targeting both TS and topoisomerase I (TopoI [10]). Activity of these targets is essential for any replicating cell regardless of driving mutations. In our previous analysis, Jurkat cells, a human T cell ALL line, exhibited low nanomolar sensitivity to F10 suggesting F10 may also have utility in ALL. In this study we sought to determine the activity, toxicity and uptake of F10 in preclinical models of ALL.

## RESULTS

**Figure 1:**
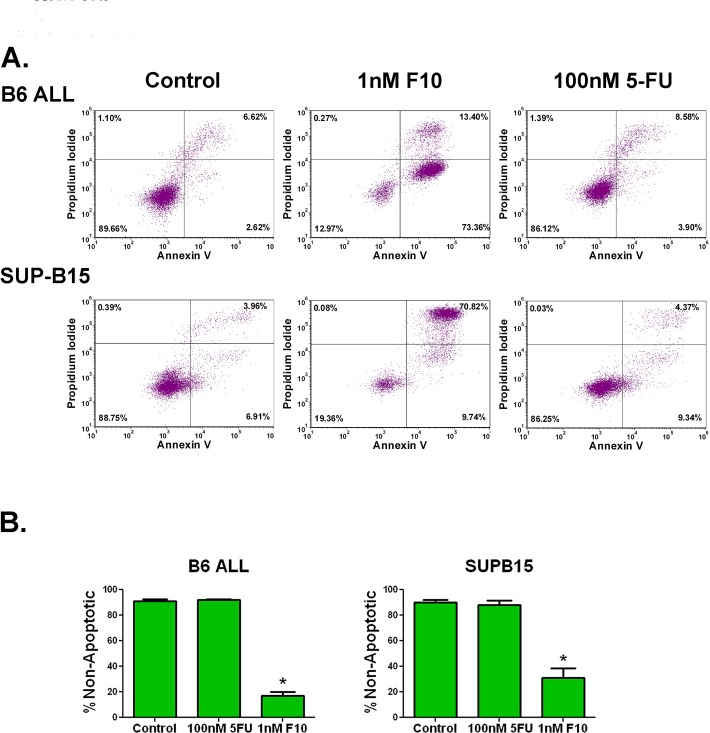
F10 exposure induces apoptosis in ALL cells A) Flow cytometry of Annexin V assay. B6 ALL or SUP-B15 ALL cells were exposed to the indicated amounts of F10 or 5-FU for 72 hours and assessed for apoptosis by annexin V and propidium iodide staining. B) Quantitation of non-apoptotic cells. Quantitation of the above from 3 independent experiments. 5-FU treatment was not significantly different from controls. *=p value <0.001.

F10 is highly active against ALL *in vitro*. In our previous work, F10 demonstrated high-potency cell killing in the human T cell ALL line, Jurkat. To assess its activity further, a panel of human and mouse ALL cell lines were exposed to a titration of F10, 5-FU, cytarabine, or doxorubicin. After 72 hours, viability was assessed and the IC_50_ values for each were determined in three separate experiments each done in triplicate. The average IC_50_ value of F10 for all cell lines was 1.48 nM (range 0.07 to 5.4 nM). In all cases, F10 was the most potent agent tested, and for all cell lines tested, it was more than 1000 times more potent than 5-FU (Table 1). In AML, F10 exposure results in a robust induction of apoptosis [10]. To see if ALL cells respond in a similar fashion, we exposed murine and human ALL cells to F10 and assessed induction of apoptosis. After 72-hour exposure, there was a robust induction of Annexin V and PI positive cells when treated with 1nM F10 that could not be equalled by 100nM 5-FU despite 10 times more fluoropyrimidine content (Figures 1A, 1B, Supplemental Figure 1). These results demonstrate that F10 is handled as a distinct biochemical entity and is not just another vehicle to deliver 5-FU.

**Table 1:** Mean IC_50_ Values for F10, 5-FU, Doxorubicin and Cytarabine

Treatment	B6 ALL	DG75	Molt-4	SUP-B15	CCRF-CEM
F10	0.07 nM (0.06 to 0.08)	4.12 nM (2.79- 6.10)	1.88 nM (1.74- 2.04)	0.21 nM (0.20- 0.22)	1.14 nM (0.82- 1.58)
5-FU	799 nM (632- 1,010)	13,980 nM (7,425- 26,310)	4,963 nM (3,933- 6,262)	425.3 nM (117.7-1537.0)	>50,000 nM
Doxorubicin	4.84 nM (3.98-5.90)	150.3 nM (131.7- 171.5)	19.48 nM (18.0- 21.1)	19.40 nM (16.6-22.7)	36.29 nM (21.7-60.7)
Cytarabine	11.93 nM (12.8- 15.2)	64.28 nM (41.7- 99.0)	10.63 nM (9.7- 11.6)	ND	8.32 nM (7.73- 8.96)

95% confidence intervals are shown in the parenthesis.

Cells were subjected to 72 hour exposures prior to viability.

ND=Not Determined

**Figure 2:**
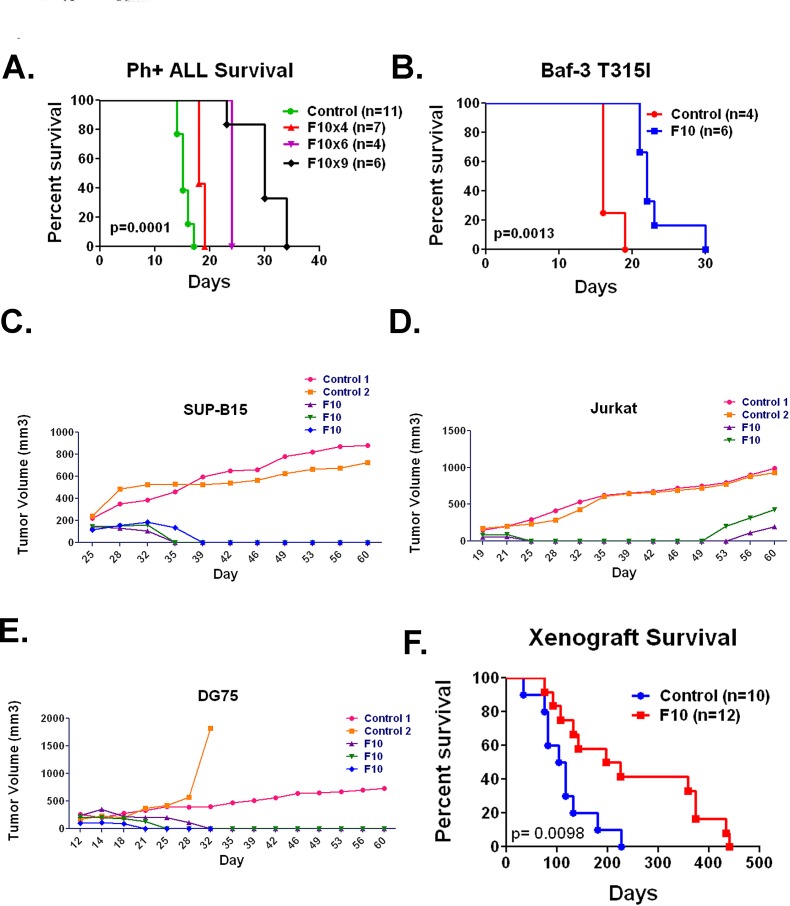
F10 is active against multiple ALL models *in vivo* A) Survival of C57Bl/6 mice injected with B6 ALL cells and treated with saline or F10 at 300 mg/kg as indicated. B) Survival of Balb/c mice injected with Baf-3 cells expressing the T315I variant of BCR-ABL treated with saline or F10 at 300 mg/kg QOD x4 doses as indicated. C) Volumes of SUP-B15 tumors in nude mice treated with saline or F10 at 300 mg/kg QOD x5 doses as indicated. D) Volumes of Jurkat tumors in nude mice treated with saline or F10 at 300 mg/kg QOD x5 doses as indicated. E) Volumes of DG-75 tumors in nude mice treated with saline or F10 at 300 mg/kg QOD x5 doses as indicated. F) Survival of all xenograft bearing mice from time of injection treated with saline or F10 at 300 mg/kg QOD x5 doses as indicated. All p values were derived from log rank tests.

F10 is highly active against multiple preclinical ALL models *in vivo*. F10 exhibited high potency against ALL cells *in vitro* however many variables present in patients are not accounted for in these types of assays. In order to be of clinical utility F10 must be able to induce ALL cell death when the cells are in the appropriate bone marrow microenvironment, with intact immune systems and with transient exposures. The precise syngeneic B cell ALL model (B6 ALL) incorporates all of these factors [11]. We injected 1×10^6^ B6 ALL cells into 6-8 week old syngeneic C57Bl/6 mice. After confirmation of engraftment by luciferase imaging mice were treated with F10 at 300 mg/kg or saline by tail vein injection every other day (QOD) for 4, 6 or 9 treatments. After 4 treatments, F10 significantly prolonged survival compared to controls (median OS 15 vs 18 days, p= < 0.0001). By 6 treatments, the median OS increased to 24 days (p= 0.0009), and by 9 treatments, F10 had doubled the median OS of controls at 30 days (p= 0.0001, Figure 2A).

To ensure these effects were not secondary to some inherent sensitivity in the B6 ALL cell line, we tested Baf-3 cells infected with an MSCV-based virus that expressed the T315 variant of BCR-ABL seen in many cases of relapsed Philadelphia chromosome-positive ALL [8]. Infected cells were then injected into Balb/c mice. Once engraftment was confirmed by bioluminescent imaging, mice received F10 at 300 mg/kg or saline by tail vein injection QOD × 4 doses. As before, F10 treatment significantly prolonged survival (p= 0.0013; Figure 2B).

To establish that the high efficacy was not limited to murine ALL cells, we tested F10 in SUP-B15, Jurkat, and DG75 human ALL cell lines in xenograft models. Nude mice were subcutaneously injected with 2×10^6^ cells in Matrigel. Once a reproducibly palpable tumor was established, mice were treated with F10 at 300 mg/kg or saline by tail vein injection QOD for 5 doses. In all cases, F10 treatment resulted in tumor regression (Figure 2C, D, E), and in several cases resulted in complete eradication of the tumor. F10 also resulted in a significant survival advantage (p= 0.0098; Figure 2F). These data demonstrate the robust in vivo antileukemic activity of F10 and further show its activity against ALL with diverse lineages and driving mutations.

**Figure 3:**
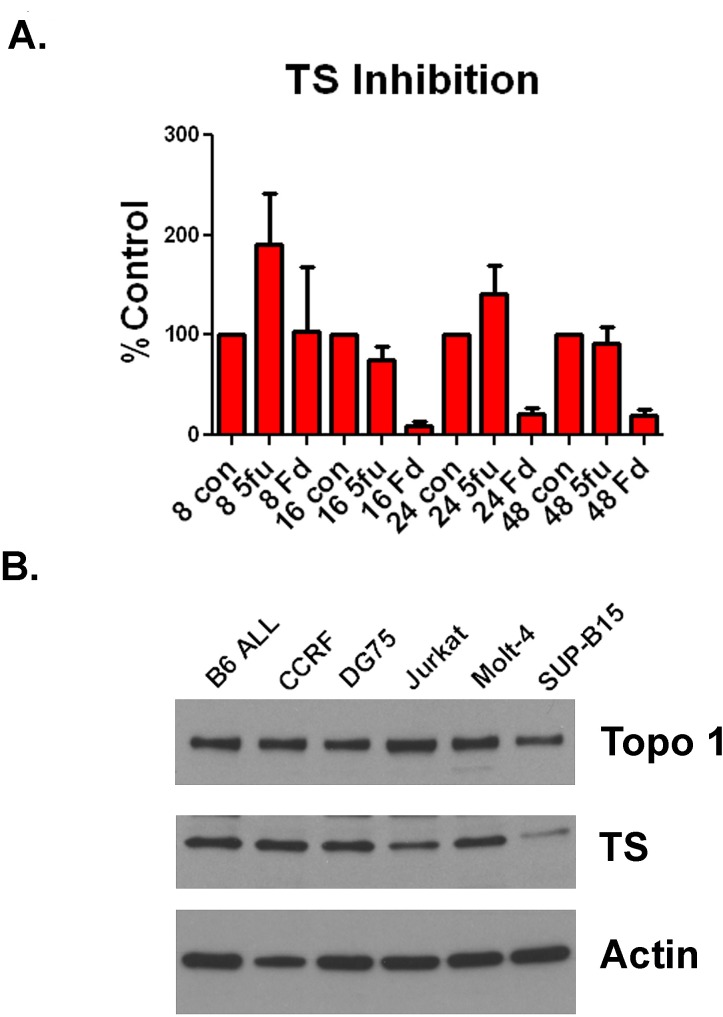
F10 is a potent inhibitor of TS and ALL cells express TS and TopoI (A) TS inhibition assay. Jurkat cells were exposed to 10nM F10 (Fd) or 100nM 5-FU (5-FU) for either 8, 16, 24 or 48 hours as indicated. Cells were then lysed and assayed for TS activity. Activity is plotted as percentage of control. B) Western blot. ALL cells were lysed and extracts blotted for TS, TopoI or actin as indicated.

F10 is a potent inhibitor of TS and TS and Topoisomerase I are widely expressed in human ALL cells. Previously, we demonstrated that F10 was a more potent inhibitor of TS than 5-FU in human AML cells [10]. To determine if F10 had a similar inhibitory activity in ALL cells, we treated Jurkat cells with either 10 nM F10 or 100 nM 5-FU. As in AML, F10 treatment resulted in profound and prolonged TS inhibition that could not be achieved by 5-FU despite the identical amount of fluoropyrimidine being present (Figure 3A). Additionally, F10 generates trapped topoisomerase I (topoI) cleavage complexes resulting in apoptosis [12]. To determine how widely expressed TS and TopoI are in ALL cells, we performed Western blots against both proteins in a panel of human and murine ALL cell lines. As expected for proteins required for DNA synthesis, both were detectable in all cell lines, despite their diverse linages and driving mutations (Figure 3B). These data demonstrate that F10 is a potent inhibitor of TS, and that TS and TopoI are widely expressed in ALL. Taken together, these data suggest that F10 is likely to be efficacious for many patients with ALL.

**Figure 4:**
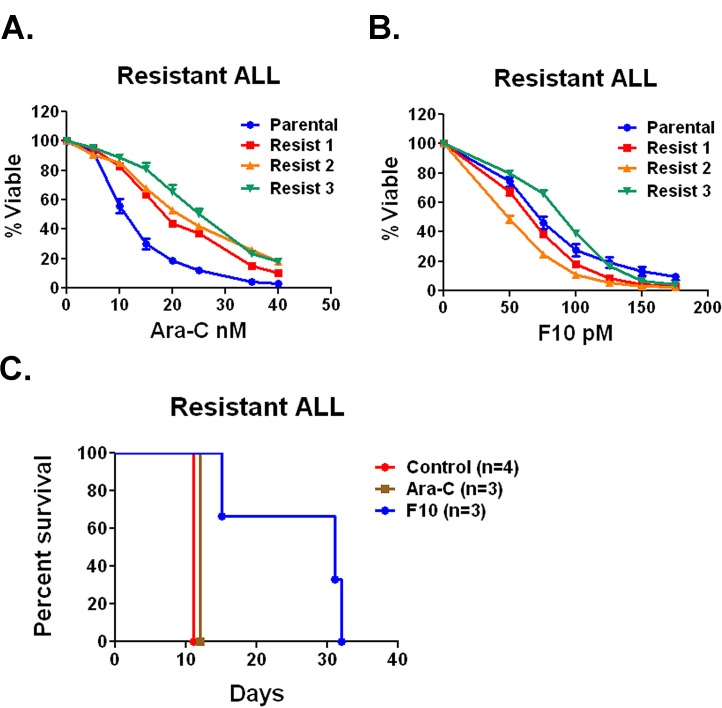
F10 has activity against Ara-C resistant ALL *in vivo* and *in vitro* A) Cell viability assays. Parental B6 ALL cells or Ara-C resistant lines were treated with a titration of Ara-C for 72 hours and viability was assessed. B) Parental B6 ALL cells or Ara-C resistant lines were treated with a titration of F10 for 72 hours and viability was assessed. C) Survival of C57Bl/6 mice injected with Ara-C resistant B6 ALL cells treated with saline, F10 at 300 mg/kg or Ara-C at 100 mg/kg QOD as indicated. p value derived from log rank test.

F10 is active in cytarabine-resistant ALL cells. Induction and consolidation therapy for ALL uses essentially all active agents. As a result, when the disease relapses response rates are low and median survival is less than a year [6]. Since F10 has distinct cellular targets, we sought to determine if it would have activity against ALL cells that acquired resistance to cytarabine. Cytarabine was chosen because it is widely used in ALL treatment. To generate cytarabine-resistant cells, we injected C57Bl/6 mice with B6 ALL cells and treated them with cytarabine until loss of response. When the mice were moribund, we harvested bilateral femur cells from 3 separate mice and placed them in culture. The relapsed cells were resistant to cytarabine compared with the parental cell line with a more than doubling of the IC50 from 10.9 nM to an average of 24.7 nM (Figure 4A). In contrast, the average IC50 value for F10 in the resistant lines was 70.07 pM comparable to the parental line IC50 of 68.95 pM (Figure 4B).

We then injected C57Bl/6 mice with relapsed cells; after engraftment, we treated mice either with saline, cytarabine (100 mg/kg QOD), or F10 (300 mg/kg QOD) and followed them for survival. Saline-treated mice died in a median of 12 days and cytarabine-treated animals lived only a day longer. F10-treated animals lived for a median of 31 days, comparable to the survival achieved with the parental cell line (Figure 4C). These data demonstrate that F10 is not cross-resistant with cytarabine and suggests it will be highly efficacious in relapsed ALL.

**Figure 5:**
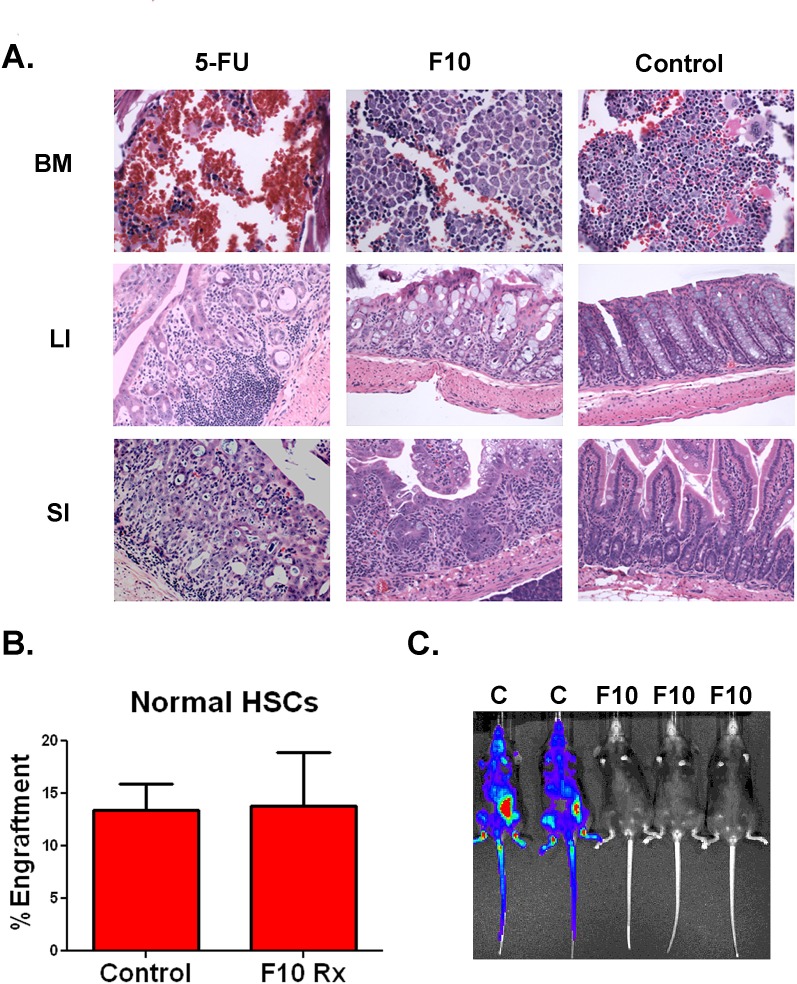
F10 is well tolerated and does not injure human HSCs A) Hematoxylin and eosin staining of organs from Balb/c mice treated with either saline, F10 at 300 mg/kg or 5-FU at 121 mg/kg QOD x4 as indicated. Animals were sacrificed 72 hours following their last dose. BM=bone marrow, LI=large intestine, SI=small intestine. B) Engraftment of human HSCs. Human HSCs were treated with saline or 50nM F10 for 24 hours as indicated and then injected into NSG mice. After 60 days mice were euthanized and bilateral femur cells were assayed for engraftment. C) Bioluminescence of C57Bl/6 mice injected with B6 ALL cells treated with saline (C) or 50 nM F10 (F10) for 24 hours.

F10 is well tolerated and does not harm human hematopoietic stem cells. In previous studies, F10 was well tolerated by C57Bl/6 mice [10]. To establish that F10 tolerability was not specific to the C57Bl/6 strain, we treated Balb/c mice with saline, 5-FU (121 mg/kg) or F10 (300 mg/kg QOD x4) and then sacrificed animals 72 hours after the last dose. The 5-FU dose was chosen to deliver the same amount of fluoropyrimidine as the F10 dose at 300 mg/kg. Tissues from the gastrointestinal tract and bone marrow were analyzed by a veterinary pathologist blinded to treatment. F10 treatment resulted in only minimal marrow toxicity, whereas 5-FU resulted in a pancytopenic and hemorrhagic marrow (Figure 5A). Similarly, 5-FU treatment induced abundant tissue damage and immune cell infiltration in the gastrointestinal tract, whereas F10 treatment resulted in only a mild increase in apoptotic cells (Figure 5A).

Previous work has demonstrated that F10 does not significantly alter the ability of murine HSCs to engraft into recipients compared to control cells [10]. To determine if F10 would inhibit the ability of human HSCs to engraft, we treated normal human HSCs with saline or 50nM F10 for 24 hours and then injected the cells into NSG transgenic mice. After 60 days mice were sacrificed, bilateral femur cells harvested and engraftment assessed by staining for human CD45. Pretreatment with 50 nM F10 did not alter the engraftment of human HSCs (Figure 5B). By contrast, when B6 ALL cells were treated with saline or 50 nM F10 for 24 hours before injection into syngeneic C57Bl/6 mice, F10 treatment significantly reduced engraftment (Figure 5C). Additionally, F10 protected the mice from leukemia-induced weight loss during treatment and for several days after (Supplemental Figure 2). These data demonstrate that F10 has little toxicity towards human HSCs, bone marrow, and the gastrointestinal tract whereas it is highly potent against murine ALL-initiating cells.

**Figure 6:**
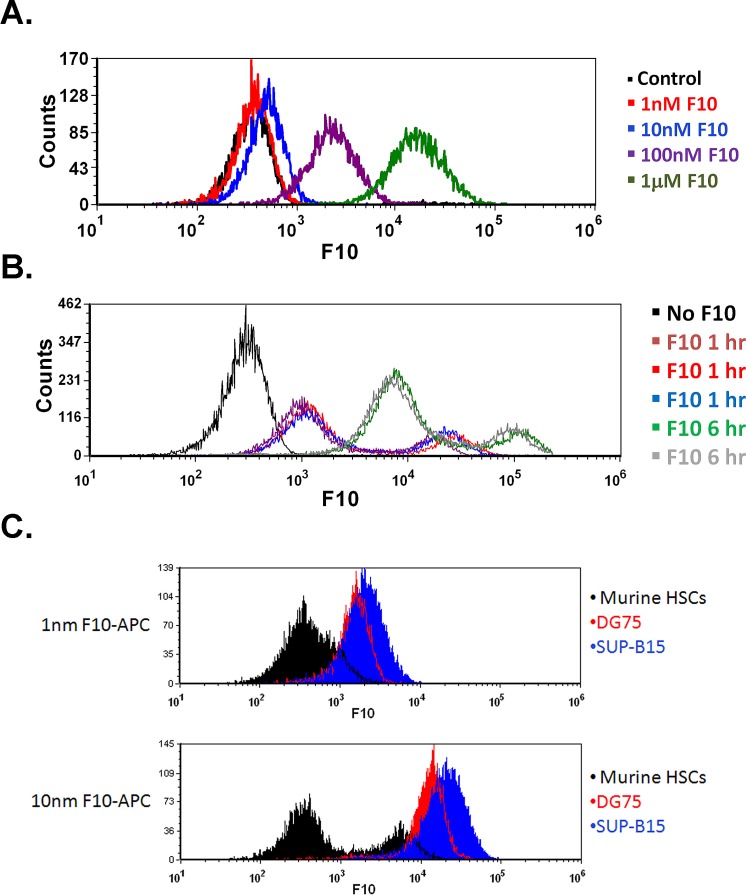
F10 is rapidly taken up by ALL cells *in vitro* and *in vivo* A) SUP-B15 cells were incubated with the indicated amounts of Quasar 670-labeled F10 for 30 minutes and assayed by flow cytometry. B) In vivo uptake of Quasar 670-labeled F10. Moribund mice injected with B6 ALL cells were treated with 2 mg/kg Quasar 670-labeled F10 and bilateral femur cells were harvested after 2 or 6 hours as indicated. C) ALL cells take up F10 more rapidly than normal murine HSCs. DG75, SUP-B15 or lineage depleted femur cells from C57Bl/6 mice were treated with the indicated amount of Quasar 670-labeled F10 for 2 hours and assayed by flow cytometry.

F10 is efficiently taken up by ALL cells *in vitro* and *in vivo*. Previous studies demonstrated that leukemia cells take up oligonucleotides with greater efficacy than normal white blood cells [9]. To determine the uptake characteristics of F10, we incubated human ALL cells with a fluorescently labeled F10. When DG75 and SUP-B15 cells were incubated with Quasar 670-labeled F10, mean fluorescence intensity increased rapidly, with detectable uptake in as little as 30 minutes (Figure 6A). Furthermore, this uptake was diminished in the presence of an excess of unlabeled F10 and had profound temperature dependence, suggestive of an active carrier-mediated process (Supplemental Figure 3A+B). To confirm rapid uptake occurred in vivo, we injected highly leukemic C57Bl/6 mice with Quasar 670-labeled F10 and harvested bilateral femur cells after 2 and 6 hours. Uptake was detectable after 2 hours and increased at 6 hours (Figure 6B). To assess if the differential toxicity seen between normal marrow cells and ALL cells was related to uptake, we incubated linage-depleted murine marrow cells and DG75 and SUP-B15 cells with Quasar 670-labeled F10. Both ALL cell lines had a more rapid uptake of F10 compared to the linage-depleted marrow cells (Figure 6C). These data demonstrate a rapid uptake of F10 by ALL cells compared to normal hematopoietic cells. This differential uptake may contribute to the high efficacy and low toxicity of F10.

## DISCUSSION

ALL is an aggressive cancer of the marrow resulting in marrow failure and death. Despite major advances in the cure rate of ALL in children, results in adults remain unsatisfactory. The current drug development paradigm in oncology is heavily weighted towards drug targets that are uniquely expressed by tumor cells. The belief is that specific inhibition of such targets will result in cancer cell death while sparing normal cells. While highly effective in genetically homogenous malignancies in complex malignancies, like the acute leukemias, such targeted therapy simply selects for resistant subclones. Here we describe a different approach where the drug targets are absolutely required for cell proliferation regardless of driving mutations. In this paradigm, agents are judged by their degree of selective uptake, not the selective expression of the target.

F10 is a poison oligodeoxynucleotide that has remarkable antileukemic activity against multiple preclinical AML models [10]. We observed in those studies that F10 was also highly effective against a human T cell ALL line. Additionally, leukemia cells have enhanced uptake of deoxy-oligonucleotides [9]. For these reasons we sought to determine the efficacy of F10 against preclinical ALL models.

The current report includes several important observations. First, F10 was highly effective against both murine and human ALL cells, with low nanomolar to picomolar IC_50_ values. It also demonstrated in vivo efficacy against 2 separate syngeneic and 3 xenograft ALL models. Second, the targets of F10, TS, and TopoI, are widely expressed in ALL cell lines. F10 displayed potent and prolonged TS inhibition in ALL cells that could not be equaled by 5-FU. Third, F10 had remarkable activity against ALL cells that were resistant to cytarabine both in vitro and in vivo, demonstrating a lack of cross resistance between the agents. Fourth, F10 was very well tolerated by Balb/c mice. Importantly, it did not affect the ability of normal human HSCs to engraft in immunocompromised mice, but it severely impaired ability of murine ALL cells to engraft. Finally, F10 was rapidly taken up by ALL cells in a highly temperature- dependent fashion that was inhibited in the presence of an excess of unlabeled compound, suggesting receptor mediated active transport. Further studies to characterize the uptake mechanism of F10 are ongoing.

F10's unique mechanism of action results in incorporation of dUMP and FdUMP into DNA in actively replicating cells [12]. As a result, once cells have completed S phase, they are can no longer be rescued by exogenous thymidine and undergo apoptotic cell death [12]. This apoptotic response does not appear to be p53-dependent [10]. These characteristics suggest F10 may have activity in hard-to-treat ALL subsets with loss of p53 [13]. Additionally, F10 and its metabolite FdUMP are chemically distinct from other nucleoside analogs used to treat ALL, and should not be subjected to dephosphorylation by cytosolic 5'-nucleotidase II (NT5C2). This is of interest as activating mutations in NT5C2 have been found in 20% of relapsed T cell ALL patients [14].

The use of therapeutic oligonucleotides to target specific genes has met with some success in the treatment of leukemia [15-17]. F10 has several advantages compared to this approach, because it directly targets TS and TopoI without the need of base pairing to its target or shRNA processing. The functions of F10's targets are essential for cell replication, regardless of driving mutations. This is in contrast to targeting antiapoptotic or driving oncogenes that could result in the outgrowth of resistant sub-clones. It is of interest that the most sensitive human ALL cell line was SUP-B15 and this line expressed TS at the lowest level of all cell lines suggesting an inverse correlation between TS expression level and F10 sensitivity. This is consistent with our previous work in AML where cells overexpressing TS were selected for by F10 treatment [10]. If this relationship is confirmed, baseline TS levels could be used as a predictive marker for future clinical trials.

In summary, F10 exhibits remarkable activity against human and murine ALL cells in vitro and in vivo by inhibition of TS and subsequent induction of apoptosis. F10 rapidly internalized by ALL cells and has low toxicity, favorable chemical properties and straight forward synthesis. It is an ideal candidate for future clinical development, particularly as an agent that can be used in developing countries. These data demonstrate agents that target “final common pathways” but with differential uptake can be safe and effective, even against genetically complex and aggressive leukemias.

## METHODS

### Cell culture and viability assays

B6 ALL cells were a kind gift of Dr Nidal Boulos (St. Jude Children's Research Hospital, Memphis, TN [11]). All human cell lines were maintained in RPMI media (Gibco, Carlsbad, CA) supplemented with 10% FBS, penicillin and streptomycin. Cells were grown at 37°C with 5% CO_2_. All murine cells were maintained in B cell media (45% DMEM, 45% IMDM, 10% FBS, supplemented with penicillin and streptomycin). Viability assays were done using the Cell Titer-Glo assay (Promega, Madison, WI) according to the manufacturer's protocol or by Trypan blue exclusion assay using the Countess cell counting system (Invitrogen, Carlsbad, CA). All viability assays were done in triplicate in at least three separate experiments (at least 9 values for each concentration tested).

### Normal human HSCs

All samples were collected under an IRB-approved protocol. All patients gave written informed consent. All samples were obtained during clinically-indicated procedures. HSCs were obtained from healthy allogeneic stem cell transplant donors. Cells were obtained from GM-CSF-primed apheresis of peripheral blood, Ficoll separated, and stored at -80°C until use.

### Western blots

Samples were lysed in Laemmli buffer, separated by SDS-PAGE, and transferred to an Immobilon PVDF membrane (Millipore, Billerica, MA). Antibodies against p53 (IMX25, 1:1000; Leica Microsystems), TS (#35-5800, 1:1000; Invitrogen), Topoisomerase I (556597, 1:2000; BD Pharmingen), Caspase 3 (9662, 1:2000; Cell Signalling) and actin (AC-15, 1:5000; Abcam) were used. Secondary antibodies were anti-mouse (7076, 1:5000; Cell Signalling) or anti-rabbit (7074, 1:5000; Cell Signalling).

### In vivo treatment studies

The Wake Forest University Institutional Animal Care and Use Committee approved all mouse experiments. Luciferase-tagged B6 ALL leukemia cells were transplanted into 6- to 8-wk-old, female C57Bl/6 or Balb/c recipient mice by tail-vein injection of 1 × 10^6^ viable cells for parental studies or 0.5 × 10^6^ for relapsed studies. Mice were monitored by bioluminescent imaging on day 6. Imaging was performed using an IVIS100 imaging system (Caliper LifeSciences, Hopkinton, MA). Mice were injected with 150 mg/kg D-Luciferin (Gold Biotechnology, St. Louis, MO), anesthetized with isoflurane, and imaged for 2 min. For the xenograft studies, 6-8-wk old nude mice were subcutaneously injected with 2×10^6^ cells mixed with 200 mcl matrigel (BD Bioscience). Chemotherapy was initiated upon detection of clear signals or reproducibly palpable flank tumors. Mice were treated with 300mg/kg F10 by tail vein injection. Control animals were injected with PBS. Tumor volumes were calculated using the method of Jensen et. al. [18]. Repeat luciferase imaging or tumor volume measurements were performed following treatment. In vivo uptake assays were done by injection of parental B6 ALL cells or saline as above and animals were monitored until body-wide bioluminescence was detected with areas of saturation consistent with high tumor burden. Animals were then injected with 1-2 mg of Quasar 670-labeled F10 and after 2 hours animals were euthanized and bilateral femur marrow isolated and analyzed by flow cytometry.

### Generation of cytarabine resistant ALL cells

Luciferase-tagged B6 ALL leukemia cells were transplanted into 6- to 8-wk-old, female C57Bl/6 recipient mice by tail-vein injection of 1 × 10^6^ viable cells. Upon detection of engraftment by bioluminescent imaging mice were treated with cytarabine at 100 mg/kg until they became moribund. Animals were then humanely euthanized and leukemia cells harvested from femur marrow. Three separate animals were harvested and the resulting cells were assessed for cytarabine resistance. These cells were then grown in culture and injected into secondary recipients as above.

### Toxicology studies and murine bone marrow transplantation

Normal Balb/c mice were treated with the identical dose, schedule and route of each drug as in the efficacy studies (i.e. Day 1, 3, 5 and 7). 72 hours after the last dose, animals were sacrificed, bilateral femoral cells harvested and organs fixed in 10% neutral buffered formalin followed by routine tissue processing and sectioning for hematoxylin and eosin staining. Slides were then reviewed by a veterinary pathologist using a Nikon Eclipse 50i light microscope. Tissues were photographed using the NIS Elements D3.10 camera and software system. For transplant studies transgenic NSG (Jackson Laboratories) mice were irradiated to 2.7 gray and injected with 2.6×10^6^ normal human HSCs. After 60 days recipient femoral bone marrow was harvested and stained with APC-conjugated anti-human CD45 antibody (BD Pharmingen, San Diego, CA) and analyzed by flow cytometry.

### Apoptosis assays

Cells were seeded in 6-well plates at 25,000 or 50,000 cells/ml in 3mls, grown for 2 days and treated with the indicated drug. After centrifugation and washing in cold PBS cells were stained with PI (Sigma Aldrich, St. Louis, MO) and APC-conjugated Annexin V in a binding buffer (10 mM HEPES (pH 7.4), 140 mM NaCl, and 2.5 mM CaCl2 solution) (BD Pharmingen, San Jose, CA) according to the manufacturer's protocol. Flow cytometric analysis was conducted on a Accuri flow cytometer (BD Biosciences).

### F10 Uptake assays

Cells were incubated for the indicated amount of time with F10 labeled with Quasar 670 (Biosearch Technologies Novato, CA) at the 5'-terminus. All in vitro incubations were done at 37 °C in 5% CO_2_. The fluorophore was incorporated as a phosphoramidite during solid-phase DNA synthesis.

### TS catalytic activity

Cells were plated at 1.5 x10^6^ cells in 100 mm^2^ plates and grown overnight in RPMI 1640 medium with 20% FBS. Cells were exposed to the indicated drug for 8, 16, 24, or 48 hours. Cells were then harvested in 25 mM Tris-HCl, pH 7.4 with Complete Protease Inhibitor Cocktail (Roche), put through two freeze/thaw cycles, and vortexed. The lysates were centrifuged at 10,000 × g for 10 minutes at 4°C. TS assays were performed in a final volume of 200 μl containing 75 μM 5,10 methylene tetrahydrofolate in 0.5 M NaOH (Schircks Laboratories, Switzerland), 10 μM dUMP, 200,000 dpm of 3H-dUMP (Moravek Biochemicals), 100 μM 2-mercaptoethanol, and 25 mM KH2PO4, pH 7.4. Cell lysate (400 μg of protein) was added to the reaction buffer. Reactions were incubated at 37°C for 30 minutes and stopped by addition of 100 μl of 20% TCA, incubated for 5 minutes on ice. 200 μL of activated charcoal solution (10 g activated charcoal, 0.25 g BSA, 0.25 gm dextran sulfate, in 100 ml of water) was added, vortexed and maintained at room temperature for 10 minutes. Reactions were centrifuged at 10,000 × g for 30 minutes. 200 μL aliquots of the supernatant were read by scintillation counting. All reactions were repeated a minimum of three times.

### Statistical analysis

All means were compared by two-tailed Student's T test. Overall survival (OS) curves were estimated by the Kaplan-Meier method and p values were determined by the log rank test. P values below 0.05 were considered significant. Analysis was performed using Graph Pad Prism version 5.02 (Graph Pad Software Inc).

## SUPPLEMENTARY FIGURES


