# Supplementary material for: The poison oligonucleotide F10 is highly effective against acute lymphoblastic leukemia while sparing normal hematopoietic cells
Source: Oncotarget. 2014 May 1;5(12):4170–9. doi: 10.18632/oncotarget.1937 (PMC4147314; doi:10.18632/oncotarget.1937)
Supplement: Supplementary file 1 [file oncotarget-05-4170-s001.pdf]

The poison oligonucleotide F10 is highly effective against, acute lymphoblastic leukemia while sparing normal hematopoietic cells

Supplementary Material

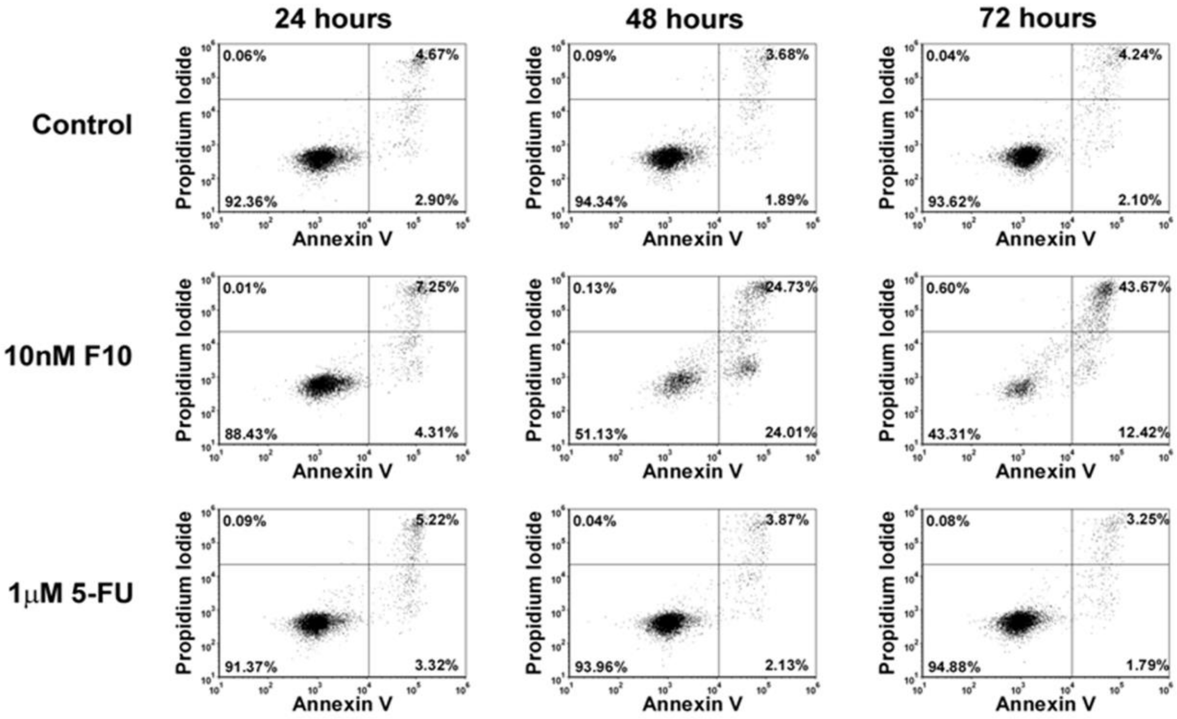

Supplementary Figure 1: F10 induces apoptosis more efficiently than 5-FU in human T cell ALL. The T cell ALL cell line Molt-4 was exposed to the indicated amount of F10 or 5-FU and then assessed for Annexin V and propidium iodide (PI) staining as indicated.

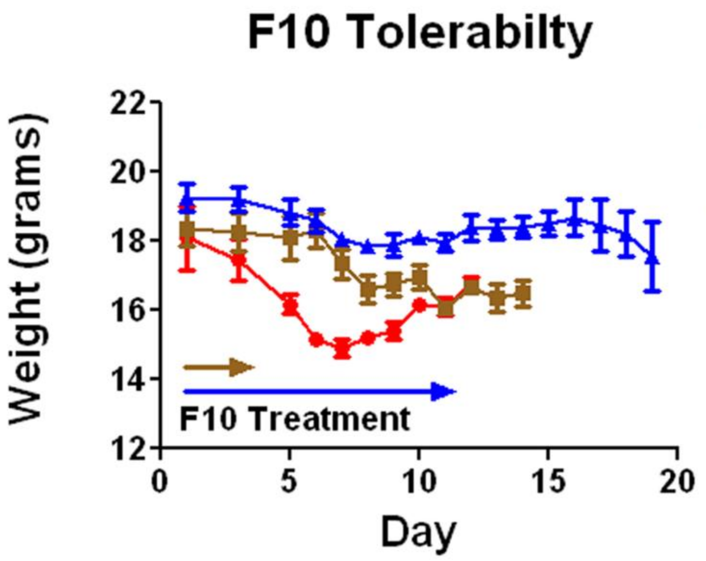

Supplementary Figure 2: F10 is well tolerated by C57Bl/6 mice. Animals were injected with B6 ALL cells and treated with saline or F10 at 300 mg/kg QOD for 2 or 6 doses as indicated and weights were followed.

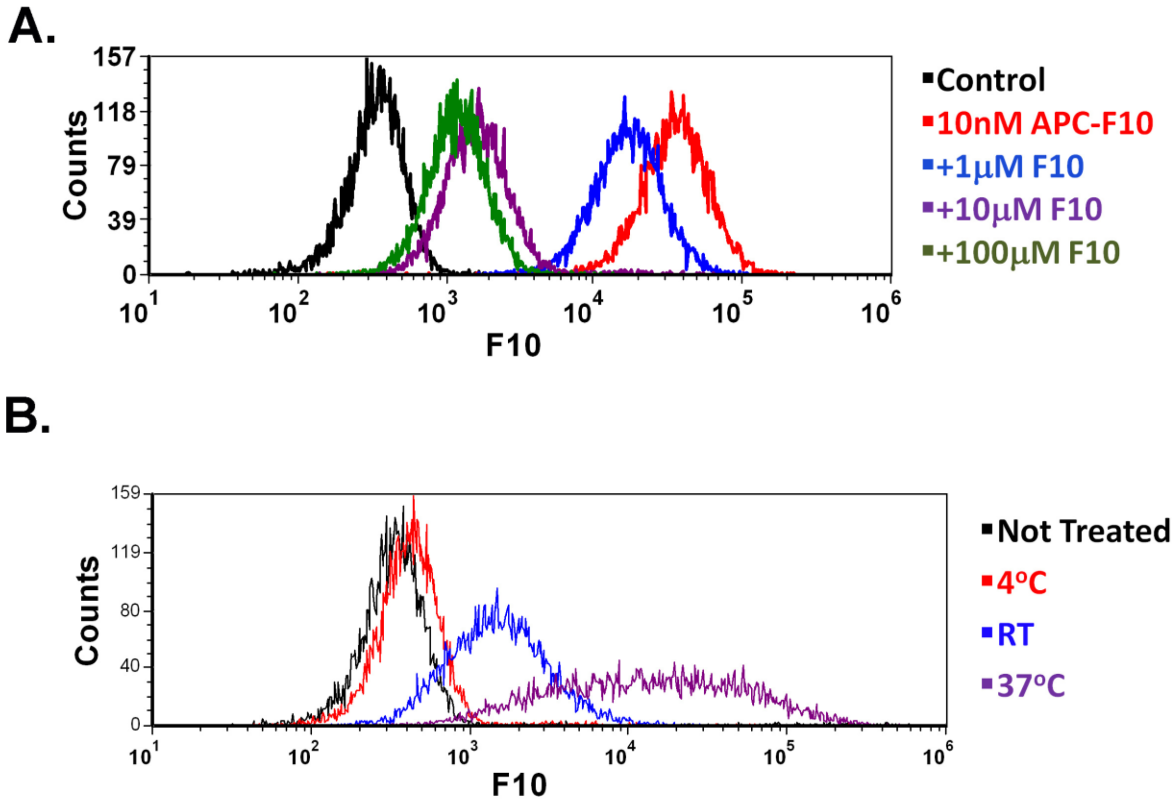

Supplementary Figure 3: F10 uptake can be competed by unlabeled drug and is highly temperature dependent. A) SUP-B15 cells were incubated with 10 nM APC labeled F10 and the indicated amount of unlabeled drug for 2 hours and then assayed by flow cytometry. B) SUB-

B15 cells were treated with 10nM APC labeled F10 and incubated at the indicated temperature for 4 hours. Cells were then assayed by flow cytometry.
